# Developing a virtual reality environment for educational and therapeutic application to investigate psychological reactivity to bullying

**DOI:** 10.1007/s10055-023-00829-5

**Published:** 2023-07-12

**Authors:** Julia R. Badger, Aitor Rovira, Daniel Freeman, Lucy Bowes

**Affiliations:** 1 Department of Experimental Psychology, University of Oxford, Oxford, UK; 2 Oxford Health NHS Foundation Trust, Oxford, UK

**Keywords:** Virtual reality, Bullying, Adolescents, Psychological reactivity, Depression, Anxiety

## Abstract

Understanding how bullying victimisation influences cognitive and emotional processes may help to direct early intervention to prevent the development of psychopathology. In a convenience sample of 67 female adolescents, we assessed the potential of a newly developed classroom-set bullying experience in virtual reality (VR) to evoke psychological reactions. Two VR experiences were co-developed with young people, one neutral and one hostile (bullying). Participants were matched and assigned to a condition based on measures of anxiety, depression, paranoia, and previous bullying, before experiencing either the neutral or hostile scenario. Before and after the VR session, participants completed measures of negative affect and levels of distress. All participants remained immersed for the whole duration, which supports the acceptability of using these VR experiences with more vulnerable participants. Those experiencing the hostile version reported greater negative affect post-immersion compared to those experiencing the neutral version (*p* = .018; *d* = 0.61). Although non-significant, a similar outcome was found regarding distress (*p* = .071; *d* = 0.37). Whilst we did not find a significant relationship between pre-existing internalisation on negative affect and distress, our sample was limited by containing adolescents with relatively low levels of previous bullying experience. Yet we still found evidence that the VR scenario evoked bullying-related psychological reactions. Further testing with a more representative groups of adolescents, especially those with more experience of bullying, would be advised. The VR scenario could potentially be used in educational and therapeutic settings to enhance empathy towards victimised children or enhance resilience following victimisation.

## Introduction

### Bullying and mental health implications

School bullying can cast a long shadow over a child’s life. Bullying, either direct or indirect, is an aggressive anti-social behaviour whereby an individual or group of individuals intentionally cause harm and upset to another individual who has less power than themselves (Olweus 1996). Worldwide data estimates that 30–35% of adolescents are involved in bullying, either as the perpetrator or the victim (Modecki et al. 2014; Przybylski and Bowes 2017), although prevalence rates differ according to the measures used. Children with high levels of internalising symptoms are more likely to experience bullying, with studies suggesting that being bullied may also, in turn, exacerbate the symptoms of anxiety (Stapinski et al. 2014; Fang et al. 2022), depression (Bowes et al. 2015; Ye et al. 2023), and psychosis (Williford et al. 2012). These symptoms can continue into adulthood. Reducing bullying continues to be a public health priority (UNICEF 2019). Whilst evidence indicates that whole-school anti-bullying programmes can successfully reduce overall prevalence rates of bullying (Vreeman and Carroll 2007; Gaffney et al. 2019; Huitsing et al. 2020) a minority of children remain victimised and may even experience increased psychopathology (Garandeau and Salmivalli 2019; Liu et al. 2021). Identifying which bullied children are most at risk of developing poor psychological outcomes is crucial for the development of targeted interventions. Understanding how experiences of peer victimisation influence real-time cognitive and emotional processes in children and adolescents would help to identify targets for early intervention to prevent the development of psychopathology.

### The potential of VR in studying bullying

For decades bullying research has been conducted using retrospective questionnaires and methodologies, which, while very valuable, have been criticised due to their reliance on accurate memory. They typically ask children and adolescents to report over a two-week to a six-month period. The over-reliance on self-reports of victimisation can be problematic; children who are bullied are also more likely to display hostile attributional biases that may lead them to misinterpret subsequent peer interactions as bullying (Williford et al. 2012). Given the increased rates of depressive symptoms among children who are bullied, there is also a greater likelihood of negative recall bias compared to children without depressive symptoms which might lead to reporting biases (Williford et al. 2012). Whilst peer nominations go some way to resolve this challenge, they are often limited to specific class or year-groups, and thus may not identify bullying outside of these contexts. To complement this work, it would also be helpful to have specific, reliable, ecologically valid and real-time methodologies. Virtual reality (VR) offers the opportunity to study bullying in a safe and controlled environment, whilst still providing a strong experience for the participant of being in a bullying situation, and enabling researchers, teachers or therapists to choose a scenario to best suit individuals with varying levels of previous bullying experience and/or mental health. Powerfully and uniquely, the participant can take on the first-person perspective within the virtual environment (VE). It is also a method that allows for standardised and controlled manipulation, where specific stimuli can be included or removed whilst the overall environment can remain the same. This enables researchers to better understand the real-time triggers and impact of bullying without the participant being in a real-life hostile situation and without the complex interactions of a real-life environment. Participants are likely to have responses to the events depicted as comparable to a real-life counterpart and, although fully immersed, participants are able to stop the VE if they become distressed by simply taking the VR headset off. They can be fully debriefed afterwards. VR has the power to immerse someone sufficiently in a situation to evoke psychological and physiological responses that enables safe study of bullying.

### Benefits if VR works

A plausible bullying experience in VR would enable researchers to understand the impact of bullying on real-time cognitions and behavioural responses. VR could mimic and therefore expose adolescents to real-life situations in which they may never have faced: being the victim of bullying. VR has already been shown to increase empathy in healthy individuals who, in VR, assume the position of someone diagnosed with schizophrenia (Kalyanaraman et al. 2010). The effect was found to be more powerful than when the participants were tasked with reading about the condition alone. As such, this technology (with which most adolescents will be fairly comfortable), could complement teaching and open up a new dialogue of understanding and empathy. A bullying experience in VR could also support previously bullied children, or at-risk children, to build their resilience and coping strategies to avoid becoming school refusers. These VR experiences could enable adolescents to face their fears in a controlled and safe environment.

### Previous VR and bullying interventions

There have been a number of recent studies that have used VR and similar technologies to better understand bullying and promote antibullying. Sapouna et al. (2010) developed a video game-like experience in which children witness bullying in the third person and are able to guide responses. Such programmes are a useful component of anti-bullying interventions designed to promote active bystander behaviour, but are less useful for understanding adolescent’s direct responses to experiencing bullying themselves. Another study aimed to assess resilience to bullying in which the participant was exposed to a harsh and overbearing superior (Krämer et al. 2018). The study used only a hostile experience (thus precluding comparison to a neutral condition), and a very specific form of victimisation: a hostile adult-like figure. Whilst this does create a power differential, this more closely mirrors maltreatment, and does not map on to the most common form of peer-on-peer bulling reported by adolescents. Other studies, such as Ingram et al. (2019) have designed 360° videos. In this case, they were designed to be used as part of a one hour a week, six-week antibullying curriculum. A total of 118 11–13 year olds participated and when immersed in the videos, watched it from the perspective of a bystander. The results showed that being immersed in the 360° videos enhanced empathy compared to those in the control condition who experienced the antibullying curriculum without the videos. A recent study (Barreda-Ángeles et al. 2021) also created 360° videos, whereby the participant assumes the role of the victim. Testing 35 10–12 year olds they found that the bully videos were overall more likely to elicit greater self-reported arousal and negative emotion than the matched neutral videos. However, this encouraging finding is limited due to the lack of pre-VR session measures (only a measure of empathy), which means it is unclear whether the findings were a true representation of reaction in any child or adolescent, or whether psychological reactivity may differ according to participants’ pre-existing individual characteristics such as internalising, and bullying experiences. A limitation also observed in an otherwise very promising study by Gu et al. (2022) who used role-exchange playing with adolescents. A total of 234 students were immersed in virtual reality scenarios as either a victim, a bully, or as a counterbalanced role-exchange between the two. The study measured pre- and post-empathy, understanding of bullying and the impact of bullying on others and found a positive move post-VR. However, again, there was no record of psychological reaction of the individuals and no indication of levels of pre-VR bullying experience or internalising.

As evidenced above, factors such as previous bullying and internalising have not only been shown to exacerbate symptoms of mental illness which can continue into adulthood, but can also result in altered reactions to situations, such as hostile attributional biases. It is therefore important to have a pre-measure of these factors before generalisations can be made.

Finally, due to the nature of many videos (which are filmed using real school children), there may also be privacy restrictions on who can access them beyond the research team. It also means that these scenarios are restricted to what has been filmed without easy alteration (e.g., characteristics (hair colour/style, skin colour and school uniform) and audio of the characters).

Despite the growing bank of immersive experiences related to bullying, both computer-generated simulations and 360° videos, there is a clear need for an adolescent peer-on-peer bullying experience that has the potential to not only expose adolescents to a controlled bullying situation but also enable researchers to monitor, in real time, the emotional and cognitive responses to the bullying experience. Such a tool may also be useful in identifying whether factors such as adolescents’ level of internalising symptoms or previous experience of bullying may leave them more vulnerable to negative psychological reactions to bullying. In this instance, our VR experience would be suitable for use in research, educational and therapeutic situations.

### Current study

This study had three aims. Firstly, to ensure that the VR experience was acceptable to vulnerable participants with regards to being immersed for the whole duration without needing to exit early. Secondly, to assess whether the bullying (hostile) version of our VR scenario was able to evoke psychological reaction in participants in terms of self-reported distress and negative affect, and thirdly, to assess whether participants with greater a) internalising symptoms and b) levels of previous bullying exposure were more reactive to the hostile condition compared to participants with lower internalising symptoms and less bullying exposure.

We hypothesised that (1) adolescents would show greater levels of distress and more negative affect following exposure to the hostile compared to the neutral scenario, and (2) adolescents with higher levels of internalising symptoms and previous bullying exposure would experience higher levels of reaction to the hostile version compared to adolescents with lower levels of internalising symptoms and less bullying exposure, and that (3) adolescents with higher levels of previous bullying experience would display a greater reaction to the neutral scenario (a hostile attributional bias) compared to those with lower levels of previous bullying experience. If the bullying experience is able to evoke a psychological reaction from participants, then this would inform future research around using this VR experience for not only research purposes but also for educational and potentially therapeutical intervention.

## Methods

### Participants

A total of 67 female adolescents aged 11–15, from two U.K. secondary schools participated in this VR study (33 were assigned to the control condition and 34 were assigned to the hostile condition). Due to the increased likelihood of same-sex bullying, we recruited only females for these all-female avatar scenarios.

Several Oxfordshire secondary schools were approached to take part in the study. When a school agreed to take part, parents were sent information letters and had the option to opt their child out of the initial questionnaire completing session (pupils had to assent on the day). After which, parents were sent a second information letter and had to opt their child into taking part in the virtual reality session (pupils had to assent on the day). Unfortunately, further recruitment was not possible due to the COVID-19 national lockdowns and school closures.

### VR setup

We used an Oculus Rift CV1 with two Oculus sensors and an Asus laptop with an Intel i7 processor, 16 Gb of RAM memory, and a Nvidia GTX 1080 graphics card. The researcher started the experience by pressing a key on the keyboard and had the option to stop it at any time.

#### VR scenario

**Fig. 1:**
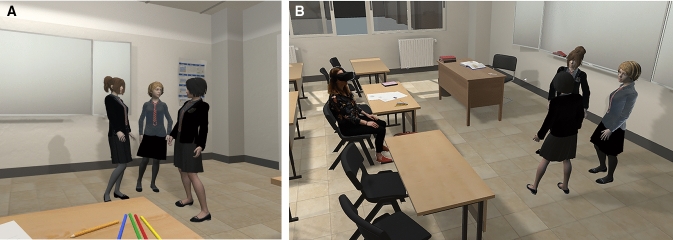
**a**. The virtual girls as seen in first person perspective from the participant’s angle. **b**. A photo composition depicting the general perspective of the classroom with the participant sitting on the first row and looking at the virtual girls

We designed a virtual classroom that looked like a typical classroom in a U.K. school. The VR scenario was built to mimic two of the most commonly reported bullying experiences among this age group; verbal and social bullying (Wang et al. 2009). The participant was sitting at a desk on the first row and there were three virtual girls standing in front of the participant and chatting between themselves (Fig. 1a and b). The conversation was either neutral for the full duration of the experience, or at some point, the girls started looking at the participant and demonstrated bullying behaviour and language, depending on the experimental condition. Both versions lasted for about 3 min.

*Neutral version* The three girls had a conversation about neutral topics such as classes they had, teachers they liked, trips to London. At no point did they engage with the participant who was sitting at a front-row desk observing.

*Hostile version* The scenario was designed to mimic low-level verbal and social bullying. The three girls started by having a neutral conversation, then one character turned her head to stare at the participant before turning back to the group and whispering and laughing. The conversation escalated to comments such as “Look at her shoes!”, “Such a loser! Does she have any friends? No. She has no friends!” and “Can’t deal with the amount of time I have to see her”. These comments were synchronised with occasional glances at the participant. We were careful that the name-calling did not cover issues that had the potential to cause undue distress in the participant, namely physical appearance (e.g., weight or skin colour). The conversation represented a private conversation between the girls, but we ensured it was loud enough so the participant could hear the majority of what was being said. Importantly, the conversation recorded for use in the VR scenario was unscripted, with the actors using their own words based on prompts we had given (i.e., to talk in a realistically mean way about the participant, but without reference to the participant’s appearance).

We simulated a power differential in our hostile bullying scenario by 1. creating three characters (numerical power advantage); 2. having the girls standing while the participant remains seated (height disadvantage), and 3) involving actors aged 15 years, at the upper end of our participant age-range (age differential). The neutral and hostile conversations were co-created with 16-year-old female volunteers, who then acted out the scene. We recorded full-body animations with an Optitrack motion capture system with 16 Flex3 infrared cameras. The audio was also recorded during the motion capture session with two wireless clip-on microphones. The scenario contained three girls, but only two were recorded at the same time. A three-minute-long idle animation for the third girl was created that complemented the two acted girls in glances and movements. The 3D animations were cleaned up and retargeted to Daz3D models in Autodesk MotionBuilder, and the audio was processed and synchronised in Audacity. The VE was built in the Unity game engine.

### Measures

Every participant completed 6 measures: The first three were used to determine a binary rating of vulnerability (higher vs. lower, cut-offs explained below): (1) the Specific Psychotic Experiences Questionnaire—paranoia subscale, (2) The Revised Child Anxiety and Depression Scale and (3) the Bullying and Cyberbullying Scale for Adolescents. Two further measures formed the pre- and post-VR experience comparison: (1) Positive and Negative Affect Schedule, (2) Subjective Units of Distress Scale and the final measure captured the participant’s immersive experience (3) VR Immersion Experience Questionnaire.

#### Specific psychotic experiences questionnaire (SPEQ; Ronald et al. 2014)

The SPEQ includes five self-report subscales designed to assess specific psychotic experiences in adolescents. This project used the paranoia subscale (15 items). This subscale requires the participant to answer a set of statements using a 6-point Likert scale. Total scores can range from 0 to 75. The average score in our sample was 11.3 (SD = 12.67). Therefore, a cut-off was determined as 11 and above = higher in paranoia (and would result in an overall score of 1); below 11 = lower in paranoia (and would result in an overall score of 0). It should be noted that this sample’s mean score is generally low compared to Fenigstein & Vanable’s (1992) original study with undergraduate students, but an average of this sample was used to create the cut-offs for a more balanced spread for this paper’s analysis. Continuous scores were used during analysis.

#### The revised child anxiety and depression scale (RCADS; Chorpita et al. 2000)

The RCADS is a 47-item self-report questionnaire designed to assess six aspects of anxiety and depression (internalising) in young people aged 8–18. The questionnaire requires participants to answer the items using a 4-point Likert scale. Total scores can range from 0 to141. To determine a binary rating of vulnerability, the total scores for subscales of generalised anxiety (possible range 0–18) and major depression (possible range 0–30) were used. The average score for anxiety in our sample was 7.3 (SD = 4.37). The average score for depression in our sample was 8.7 (SD = 6.09). Therefore, cut-offs were determined as 7 and above = higher in anxiety (and would result in an overall score of 1); below 7 = lower anxiety (and would result in an overall score of 0), and 8 and above = higher in depression (and would result in an overall score of 1); below 8 = lower depression (and would result in an overall score of 0). It should be noted that this sample’s mean scores for anxiety and depression fall exactly within the normal range for the RCADS scoring of girls of this age with higher scores falling with the raised score range. Continuous scores were used during the analyses.

#### Bullying and cyberbullying scale for adolescents (BCS-A; Thomas et al. 2019)

The BCS-A is a 13-item self-report questionnaire designed to assess the levels of experienced bullying in adolescents. It has two subscales Bullying (8 items) and Cyberbullying (5 items). The questionnaire requires participants to answer the items using a 5-point Likert scale. Total scores can range from 0 to 65. The average score in our sample was 3.03 (SD = 4.87). Therefore, a cut-off was determined as 3 and above = higher experience of bullying (and would result in an overall score of 1); below 3 = lower experience in bullying (and would result in an overall score of 0). It should be noted that this sample’s mean score is generally low but an average of this sample was used to create the cut-offs for a more balanced spread for this paper’s analysis. Continuous scores were used during the analyses.

#### Positive and negative affect schedule (PANAS; Watson et al. 1988)

The PANAS is a 20-item self-report questionnaire designed to assess positive and negative mood. It has two subscales Positive Affect (10 items) and Negative Affect (10 items). The questionnaire requires participants to answer the single word items for example ‘excited’ ‘upset’ using a 5-point Likert scale. Total scores for Positive Affect can range from 10 to 50. Total scores for Negative Affect can range from 10 to 50. This measure was used as a continuous variable.

#### Subjective units of distress scale (SUDs; Wolpe 1990)

The SUDs is a visual analogue scale measuring subjective anxiety and distress, hereafter referenced as ‘distress’. Participants rate their level of current anxiety and distress on a scale from 0 (no distress, totally relaxed), to 100 (highest anxiety/distress ever felt). Although a 1-item measure, it been found to have strong correlations with well-established longer measures of distress and show measurable change in pre- and post-measurement testing (Thyer et al. 1984). This measure was used as a continuous variable.

#### VR immersion experience questionnaire (VR-IEQ; created by the authors)

This is a self-report questionnaire that assesses the level of presence participants felt during the VR experience. Participants were required to mark their answers for each question on an analogue scale of 0–100. The four questions were summed giving an overall score range of 0–400. This overall score was divided by four, giving an average percentage variable per participant. Four items made up this VR immersion experience questionnaire with a good internal consistency: *α* = .71.

The items used were:

1. When you think back to the virtual reality experience, did it feel like you were in the classroom, or that you were watching a movie? 0 = watching a movie; 100 = in the classroom.
2. How much did it feel like the girls in the virtual reality knew you were there? 0 = not at all; 100 = absolutely.
3. How much did you think about the virtual reality headset whilst you were wearing it? 0 = all the time; 100 = forgot I was wearing it.
4. How much did you focus on the girls in the virtual reality experience? 0 = not at all; 100 = all the time.

### Procedure

Participants completed the SPEQ, RCADS and the BCS-A questionnaires on paper in their classroom two weeks before the VR experience. On the day of the VR experience, they completed the PANAS and SUDS before going into VR. After the experience, they completed the PANAS and SUDS again, along with the VR immersion questionnaire. In both experimental conditions, participants sat on a school chair with a desk in front of them. To get used to the Oculus Rift, visually explore the VE and overcome any novelty effects of being in VR, all participants first visited an empty virtual classroom for 1 min, in which they could see the virtual chair and the desk that matched the position of the furniture they were actually sitting on or by. During this time, nothing happened in the VE and the image faded out to black automatically at the end. This was also important to adjust their sight to the brightness of the VR headset and create a steady initial reaction baseline. After visiting the empty classroom, participants started either the neutral or the hostile experience, depending on the experimental condition.

### Determining study condition

Based on the data from the first three self-completed questionnaires, participants were given a binary rating for their level of each symptom of depression, anxiety, and paranoia (0 = lower and 1 = higher). Those with 2/3 or 3/3 were classified as having potentially poorer mental health and those with 0/3 or 1/3 were classified as having potentially better mental health. Three participants had missing data and were allocated randomly (1 neutral and 2 hostile); of the remaining 64 participants 32 were identified as having poorer mental health and 32 were identified as having better mental health. That binary data, along with their year group and experience of previous bullying (of which 42 had lower levels of experience and 25 had higher levels of experience), were used to match and then randomly assign participants to a condition. This ensured participants in each of the two experimental conditions were well matched.

Thirty-three were assigned to the neutral condition and 34 were assigned to the hostile condition (see Table 1 for breakdown). Participants were not informed on their condition.

**Table 1:** The assignment of participants to conditions based on year group, previous experience of bullying and level of poor mental health

	Neutral condition	Hostile condition
Year 7	13	15
Year 8	4	5
Year 9	8	8
Year 10	7	5
Lower experience of bullying	22	20
Higher experience of bullying	11	14
Lower levels of poor mental health	14	18
Higher levels of poor mental health	18	14

### Statistical analyses

In order to determine whether participants experiencing the hostile condition showed greater psychological reactivity compared to participants experiencing the neutral condition we ran two separate linear regression models, the first including distress and the second including negative affect. For the distress regression, we used post-distress as the dependent variable, condition as the independent variable and adjusted for pre-distress and year group. For the negative affect regression, we used post-negative affect as the dependent variable, condition as the independent variable and adjusted for pre-negative affect. In order to identify whether higher levels of internalising or previous experience of bullying increased reactivity to the VR scenario we repeated the above regressions and included interaction terms (condition*internalising or condition*previous bullying) as well as the covariates. All analyses were conducted in SPSS version 27.

## Results

### Change in distress and negative affect

**Fig. 2:**
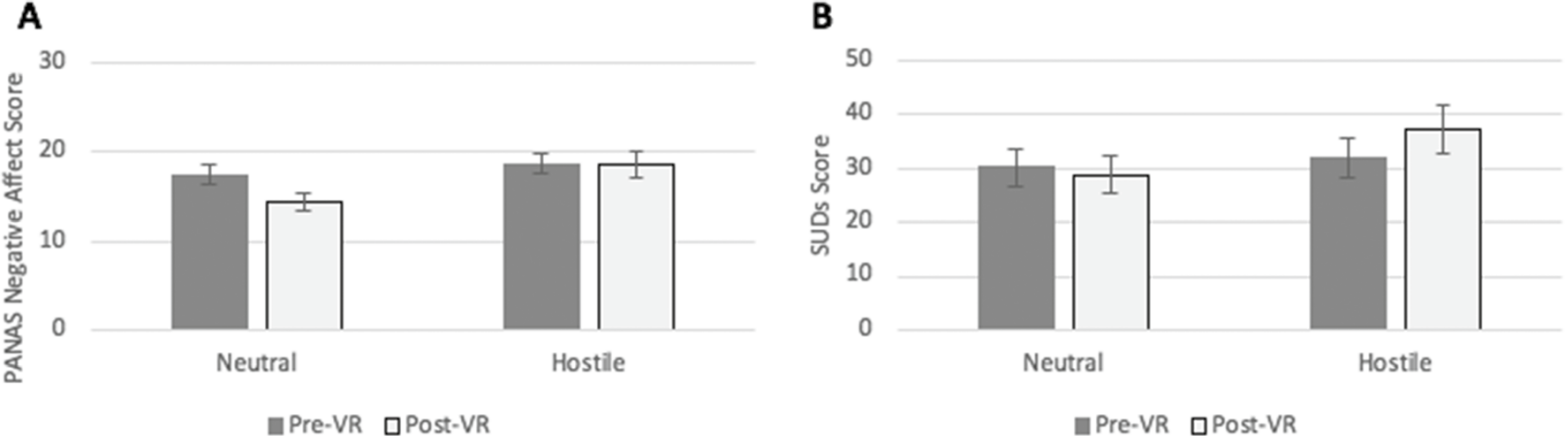
Pre- and post-VR scores for distress (left) and negative affect (right) for participants in the neutral and hostile conditions

Differences did not reach statistical significance for distress but did reach statistical significance for occurrence of negative affect (Table 3, Model 1). However, for those participants in the hostile condition, distress was higher following the VR experience (*M* = 37.26 SD = 25.64), compared to those in the neutral condition (*M* = 28.73 SD = 20.09; see Fig. 2), giving a Cohen’s *d* of 0.37. Negative affect remained high in the hostile condition (*M* = 13.38, SD = 11.63), compared to a lowering of negative affect experienced in the neutral condition (*M* = 7.39, *SD* = 7.67; see Fig. 2), giving a Cohen’s *d* of 0.61.

### Year group and the impact of VR

For both distress and negative affect, year group was significantly associated with post-VR reporting (see Tables 2 and 3). When considering the data split by year group it appears that the youngest pupils—year 7—maintained their level of negative mood regardless of condition. The year 7 pupils actually increased in their level of distress regardless of condition. It appears that the youngest year group (year 7) were more reactive to the hostile scenario, however no significant moderation by condition was observed for negative affect: *B* = − 0.79, 95% CIs = − 4.81, 0.92; *p* = .18, or distress: *B* = − 1.02, 95% CIs = − 11.62, 0.27; *p* = .06.

**Table 2:** Linear regression examining the effect of experimental condition, distress score pre-VR experience and year group, on distress score post-VR (Model 1), when controlling for previous bullying (Model 2) and internalising (Model 3)

	Model 1 (*N* = 67) B (95% CI)	Model 2 (*N* = 67) B (95% CI)	Model 3 (*N* = 67) B (95% CI)
Condition	6.47 (− 0.58, 13.52) (*p* = .071)	2.39 (− 6.18, 10.97) (*p* = .579)	− 1.77 (− 16.26, 12.73) (*p* = .808)
Distress pre	0.81 (0.64, 0.97) (*p* < .001)	0.827 (0.65, 1.00) (*p* < .001)	0.70 (0.49, 0.90) (*p* < .001)
Year group	− 3.23 (− 6.28, − 0.19) (*p* = .038)	− 2.91 (− 5.96, 0.15) (*p* = .062)	− 3.48 (− 6.57, − 0.38) (*p* = .028)
Previous bullying		− 1.38 (− 3.05, 0.29) (*p* = .104)	
Internalising			0.05 (− 0.18, 0.27) (*p* = .666)
Condition * previous bulling		1.57 (− 0.27, 3.40) (*p* = .093)	
Condition * internalising			0.22 (− 0.08, 0.52) (*p* = .154)
*R*^2^	0.62	0.64	0.63

**Table 3:** Linear regression examining the effect of VR condition, negative affect score pre-VR experience and year group, on negative affect score post-VR (Model 1), when controlling for previous bullying (Model 2) and internalising (Model 3)

	Model 1 (*N* = 67) B (95% CI)	Model 2 (*N* = 67) B (95% CI)	Model 3 (*N* = 67) B (95% CI)
Condition	4.10 (0.74, 7.46) (*p* = .018)	2.40 (− 1.71, 6.50) (*p* = .247)	− 0.28 (− 7.22, 6.66) (*p* = .935)
Negative affect pre	0.74 (0.55, 0.92) (*p* < .001)	0.75 (0.55, 0.94) (*p* < .001)	0.61 (0.37, 0.84) (*p* < .001)
Year group	− 1.77 (− 3.21, − 0.33) (*p* = .017)	− 1.65 (− 3.11, − 0.20) (*p* = .027)	− 1.82 (− 3.30, − 0.35) (*p* = .016)
Previous bullying		− 0.54 (− 1.33, 0.25) (*p* = .179)	
Internalising			0.01 (− 0.10, 0.12) (*p* = .860)
Condition * previous bulling		0.64 (− 0.23, 1.51) (*p* = .147)	
Condition * internalising			0.12 (− 0.03, 0.26) (*p* = .115)
*R*^2^	0.56	0.56	0.58

### Presence in VR

Participants felt an average total of 58% immersed and present in the VR experience overall, with those in the hostile experience feeling more immersed and present overall (65%) than the those in the neutral experience (50%). An independent t-test found this difference between conditions to be significant: *t* (65) = − 3.035; *p* = .003.

## Discussion

This study had three aims. Firstly, to ensure that the VR experience was acceptable to vulnerable participants with regards to being immersed for the whole duration without needing to exit early. Secondly, to assess whether our hostile VR scenario evoked greater distress and negative affect compared to our neutral scenario, and thirdly, to assess whether these effects were moderated by participants’ level of internalising symptoms or previous bullying exposure. These will be referenced individually below but overall, our findings broadly support the potential use of our hostile VR scenario to study responses to bullying from the victim’s first-person perspective.

In relation to our second aim, we confirmed that participants experiencing the hostile (bullying) version of our VE reported significantly greater negative affect after being immersed (using PANAS), compared to those who experienced the neutral version, with Cohen’s *d* indicating a moderate effect size (*d* = 0.61). Whilst self-reported distress was also higher in the hostile condition compared to the neutral condition, this effect did not reach statistical significance, although it had a small-medium Cohen’s *d* effect size (*d* = 0.37). These findings add support to the 2021 360° videos Barreda-Ángeles et al. (2021) study yet are also able to extend those findings by including pre-VR psychological and bullying measures: we did not find significant moderation of the effect of condition on psychological reactivity (distress or negative affect) by adolescents’ level of internalising symptoms (RCADS), or by previous exposure to bullying (BCS-A; relating to our third aim). This contradicts our second and third hypotheses and can confirm no effect of hostile attributional bias. Due to the limited sample size of this pilot study, the above vulnerability variables were not considered within one regression. Together, and in relation to our first aim, our results suggest that VR is an appropriate method for recreating a hostile social situation that evokes negative emotion, and therefore for assessing real-time reactivity during a bullying situation. There was no observed or spontaneously reported disorientation or nausea during or after the VR session.

Our findings mirrored previous literature regarding the reported positive correlation between level of anxiety and level of depressive symptoms (Cummings et al. 2014), and also the associations between previous bullying experience and symptoms of depression and anxiety (Hawker and Boulton 2000; Schoeler et al. 2018). Whilst we did not find a significant relationship between pre-existing internalisation on negative affect and distress, our small sample was limited by containing adolescents who had relatively low levels of previous bullying experience. Thus, repetition in a larger sample including adolescents with a greater level of previous bullying experience is needed.

As previously stated, VR has the capability to place individuals in challenging social situations in a safe and controlled manner before translating learned skills to the real world. Presence is an important factor to ensure ecologically valid experiences in VR and for triggering a psychological reaction in the participant. The level of presence with this sample was similar to scores reported in previous VR studies, for example Falconer (2014). In the hostile version, the scores reported were higher, again lending support to the fact that the scenarios are realistic and engaging.

The hostile scenario was designed to simulate a power differential by including three characters compared to the solo observer (numerical power advantage); by having the girls standing while the participant remains seated (height disadvantage), and by ensuring the girls looked about 15 years old—the upper end of our participant age-range (age differential). The neutral scenario mirrored the hostile scenario in every way except for the presence of bullying. This goes some way to explaining why year group was significantly associated with the post-VR scores of distress and negative affect *regardless* of condition. It appears that even in the neutral condition, this power differential was strong enough to evoke psychological reaction in our youngest year group (year 7). The effect was not observed for any of the other year groups when condition was combined.

The sample was well matched between the two conditions with regards to age, pre-existing symptoms of depression, anxiety and psychosis, and previous exposure to bullying, which provides a good insight into the effect of the VR experience rather than the sample. It also measured reactivity in adolescents using an adolescent, peer-to-peer bullying experience. Although a successful pilot of our VR scenario, there are a few limitations. Firstly, the VE only contains female characters and was tested with female participants. Although bullying does occur within the same sex, it does limit the generalisability. Future work should design both a comparable all-male scenario and also a mixed-sex scenario. The sample was also small, therefore future work should consider a larger sample which includes males as well as females, including between-sex experiences, in which a male is bullied by females and vice versa. Secondly, although the study measures psychological reactivity, future work should also consider the measurement of physiological reactivity such as skin conductance. This would allow a fuller and more detailed insight into the real-time impact of the experience on individuals using a non-self-report method. Thirdly, a measure of participants’ background exposure to gaming and/or VR would be helpful to identify any potential novelty effects on outcomes which may be masking or enhancing reactivity. Finally, mean scores for the binary rating indicate an overall low scoring of this sample on measures of anxiety, depression, paranoia and previous experience of bullying, which brings into question the representativeness of the sample to the general population. Clinical samples should be recruited alongside nonclinical samples, if the data is to be more widely applied. However, as an insight into general female samples at secondary school, this sample could be considered representative.

Despite these limitations, this study is the first known immersive VR bullying study to use peer-based experiences (both a hostile and a neutral), with the participant as the victim, whist also evoking and measuring psychological reactivity. With further work, these VEs could be used for educational or therapeutic purposes. A recent systematic review of the power of VR to change social attitudes has shown that immersive technologies outperform non-immersive technologies in relation to decreasing the distance to an abstract construal and increasing attitudinal change, for example, regarding intergroup conflict (Nikolaou et al. 2022). It is possible therefore, that these VEs could be incorporated into a classroom setting or school-based workshop to stimulate discussion around the impact of negative social peer interactions. Where Sapouna et al. (2010) used video-game like experiences to guide antibullying and promote bystander behaviours and Ingram et al. (2019) used 360° videos, our VEs could enable individuals who may have never experienced bullying first hand, to be fully immersed as a victim of bullying (in a safe and controlled way) and therefore *understand* that experience before understanding how to help prevent it. It could be an emotionally powerful learning tool. Alternatively, these VEs could be used in therapeutic situations, for example as part of therapy sessions to engage with, and support, bullying-related school refusers (children or young people who refuse to go to school), again in a safe and controlled way. Our scenarios could provide simulations to practice resilience techniques.

We have created a realistic and engaging set of VEs which can successfully be used one-to-one with adolescents. We found significant differences in the right direction in the reactivity to our neutral and hostile scenarios which were not confounded by an individual’s previous experience of bullying or their current level of internalising symptoms. However, this sample was small and limited in diversity meaning wider generalisation needs to come from additional studies. This study has shown however, that the hostile experience can be used to explore real-time reactivity to adolescent peer-on-peer bullying situations for research progression, whilst also developing a tool that could be used for both educational and therapeutic purposes. Although if used educationally or therapeutically, the impact would need to be assessed and evaluated independently. The technology is increasingly affordable and easy to set up, and it is accepted by adolescents as exciting and engaging.

## Data Availability

The datasets generated during and/or analysed during the current study are available from the corresponding author on reasonable request.
